# Participatory Ecological Assessment of Farmer Perspectives on Management of Invasive *Ageratina adenophora* in Eastern Bhutan

**DOI:** 10.1002/pei3.70110

**Published:** 2026-01-04

**Authors:** Ram Chandra Bajgai, Yadunath Bajgai, Stephen B. Johnson, Christopher Y. S. Wong

**Affiliations:** ^1^ Faculty of Forestry and Environmental Management University of New Brunswick Fredericton New Brunswick Canada; ^2^ Department of Environment & Life Sciences, Sherubtse College Royal University of Bhutan Kanglung Bhutan; ^3^ National Potato Program, National Centre for Organic Agriculture, Department of Agriculture Ministry of Agriculture and Forests Thimphu Bhutan; ^4^ Weed Research Unit, Invasive Species Biosecurity New South Wales Department of Primary Industries Orange New South Wales Australia

**Keywords:** *Ageratina adenophora*, eastern Bhutan, farmer priorities, Kruskal–Wallis test, participatory approach, sustainable management, weed control

## Abstract

*Ageratina adenophora* , a native plant to Mexico, has rapidly invaded Bhutan's landscapes from subtropical foothills to subalpine zones. This has resulted in suppressed native plant biodiversity, impacts on economically important plants, altered soil properties, and crop yield losses. Although impacted farmers managing this weed possess deep, experiential knowledge, their insights remain under‐quantified and under‐utilized. We aimed to assess farmers' perspectives on the impacts and management of *A. adenophora* in eastern Bhutan. A focus group discussion with village leaders was held in Kanglung to refine nine literature‐derived themes into five farmer‐relevant priority areas: (1) weedy characteristics, (2) growth habit, (3) competitive effects, (4) control methods, and (5) awareness. A structured questionnaire was administered to 91 randomly selected farmers to rank five sub‐themes under each priority area on a five‐point scale. Responses were analyzed using Kruskal–Wallis rank tests and weighted‐average calculations. Farmers assigned the greatest weight to characterizing *A. adenophora* as a weed (weedy characteristics) (28%), followed by control methods (24%), competition (20%), growth habit (16%), and awareness (12%). Sub‐theme rankings differed significantly within each priority area (*χ* ^2^ ≥ 78.95, *p* < 0.001). Farmers identified the species abundance, prolific seed production, and rapid seedling growth as key drivers of its aggressive spread. They perceived *A. adenophora* as both ecologically damaging and economically harmful, prioritizing management via uprooting, burning, and burying over slashing or herbicide application. By bridging the gap in quantified farmer‐perspective data through focus group discussion, structured ranking questionnaires, and non‐parametric analysis, this study uses a participatory approach for integrated invasive‐weed management. It is replicable to similar agroecological landscapes, aligning scientific strategies with local knowledge to enhance sustainable control of *A. adenophora* to protect productivity of the farming lands.

## Graphical abstract

*Ageratina adenophora* 's invasion across Bhutan threatens biodiversity and crop yields. This study quantifies farmers' experiential knowledge through participatory methods, revealing key concerns: aggressive spread, ecological harm, and preferred control strategies. Aligning local insights with scientific approaches supports sustainable weed management across similar agroecological landscapes.

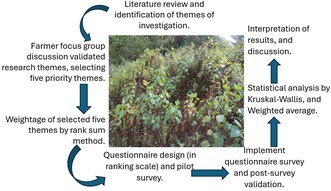

## 1 Introduction

*Ageratina adenophora* (Spreng.) R.M. King & H. Rob (Syn. *Eupatorium adenophorum* Spreng.; common name: crofton weed) (Cronk and Fuller 1995) of the Asteraceae, which is native to Mexico, is a globally invasive herbaceous plant generally found in tropical, subtropical, and warm temperate regions of the world (Yu et al. 2016; Khatri et al. 2025a, 2025b; Fartyal et al. 2025). It has spread rapidly across the globe and is now confirmed in over forty countries (Poudel et al. 2019; Fried et al. 2025). The potential of this weed to invade a diverse range of habitats, that is, heterogeneous environmental conditions, helps this weed to successfully establish and proliferate itself (Negi et al. 2025; Khatri et al. 2025a, 2025b; Fartyal et al. 2025). Instances of invasion include the United Kingdom in 1826, where it was introduced as an ornamental garden plant (Paxton 1840); Bhutan prior to 1961 (Griffith 1847; Dorjee et al. 2020) via India; and, more recently, Italy in 2013 (Del Guacchio 2013; Fried et al. 2025). Generally, *A. adenophora* flourishes in regions with high rainfall and warm temperatures (Tererai and Wood 2014). It grows in natural forests, forest margins (including rainforests), nature reserves and national parks, roadsides and waste areas, ungrazed small holdings, and cleared land (Trounce and Dyason 2003). Environments characterized by low species richness and disturbed habitats are susceptible to *A. adenophora* invasion (Wan et al. 2010). In extreme environments, it has been found at high elevations up to 3280 m above sea level (m.a.s.l) that freeze for over a month in Nepal (Siwakoti et al. 2016), and it is predicted to expand further up to 3547 m.a.s.l in the Himalayas within the next five decades under future climate scenarios (Thapa et al. 2018). In addition, Dukes (2000) pointed out that invasive plants generally respond positively to enhanced atmospheric CO_2_, especially in colder regions, which corroborates upsloping trends of *A. adenophora* with increased CO_2_ concentrations.

The invasion mechanism of *A. adenophora* has been thoroughly investigated, highlighting its phenotypic plasticity and epigenetic modification for cold resistance (Wan et al. 2010; Poudel et al. 2019; Negi et al. 2025). The high adaptability of *A. adenophora* to different environments is combined with its high reproductive capacity and diverse dispersal means (Yu et al. 2016; Fried et al. 2025). *A. adenophora* seedlings demonstrate adaptability to different soil types and fertilizers, with their high capacity to acclimate to soil conditions another key factor in successful invasion (Wan et al. 2010). *A. adenophora* can utilize plentiful nitrogen‐phosphorus‐potassium (NPK) present in the soils (Li et al. 2022; Wu et al. 2025), allowing it to outcompete the native plants (Peng et al. 2010). Zhu et al. (2011) reported that *A. adenophora* releases two primary allelochemicals, that is, 9‐oxo‐10,11‐dehydroa‐geraphorone and 9b‐hydroxyageraphorone as soil leachates (Li et al. 2010). These cause prominent crowding out effects against native plants (Li et al. 2010). Other ground‐leaching allelopathic substances such as 6‐hydorxy‐5‐isoporpyl‐3,8‐dimehtyl‐4a,5,6,7,8,8 ahexahydronaphthalen‐2(1H)‐one (HHO) and 4,7‐dimethyl‐1‐(PorPna‐2‐ylidene)‐l,4,4a,8a‐tetrahydronaphthalene‐2,6(1H,7H)‐dione (DTD) are also released (Yang et al. 2008). The volatile organic compounds (VOCs) from litter of *A. adenophora* have been reported to have a stronger suppressive effect than VOCs from native plants (Inderjit et al. 2011) with the latest findings by Wu et al. (2025) as allelochemicals being severe effectors of invasion success through allelopathy. Liao et al. (2015) suggested that leaf leachate has various effects on the seed germination and seedling growth of different plants under varying environmental conditions. Further, Jigme and Bajgai (2019) reported that *A. adenophora* leaf extract suppresses the growth of the white rust under laboratory conditions.

Invasion by *A. adenophora* leads to an increase in soil ${\mathrm{NH}}_{4}^{+}$ and ${\mathrm{NO}}_{3}^{-}$ nitrogen levels, alters the distribution of microorganisms, and diminishes total phosphorus levels (Li et al. 2022; Wu et al. 2025). These alterations create favorable conditions for *A. adenophora* to thrive in nutrient‐poor environments, facilitating its invasion while rendering native flora less competitive (Peng et al. 2010). Further, soil impacts by *A. adenophora* invasion include decreased pH levels, phosphorus deficiency, and increased concentrations of organic matter, total nitrogen, and potassium (Darji et al. 2021; Wu et al. 2025). A three‐year invasion study of the species in Sikkim, India, across elevations showed that *A. adenophora* did not show a persistent association with native flora, but the presence of other invasive species indicated that these co‐occurring invasive species must be complementary in resource use and mutually benefit from each other's presence, resulting in invasional meltdown (Verma et al. 2023).

Management of *A. adenophora* has been attempted using a variety of methods. Around the world, mechanical, cultural, chemical, and biological methods (Poudel et al. 2019) have all been found to be less than effective, and the weed continues to persist and spread. Mechanical methods such as hand uprooting, slashing, and digging/plowing are employed for control in smaller plots, but this is unrealistic for rugged terrain and larger land areas (Poudel et al. 2019). Planting more competitive forage grasses in places where the invasive weed has been removed serves as a partial solution to the problem (Wan et al. 2010). Cultural methods include harvesting biomass for livestock bedding, making biomass briquettes, using it as a substrate for growing mushrooms, and other domestic commodities (Shrestha et al. 2019). Biomass utilization practices can reduce seed production in the local area, thereby reducing its spread (Poudel et al. 2019). A double benefit of controlling invasive species and reducing CO_2_ emissions through green fuel occurs when the abundant biomass of invasive species is used to produce biofuel (Bajgai et al. 2022). There are several herbicides that kill the plant, for example, glyphosate, metsulfuron methyl, fluroxypyr, triclopyr, and 2,4‐D amine, which are applied on shoots as mentioned in Poudel et al. (2019). Several biological control agents are also used to manage *A. adenophora* , including *Baeodromus eupatorii*, *Dihammus argentatus*, *Doryluss orientalis*, *Oidaematophorus beneficus*, *Passalora argentinae*, *Procecidochares utillis*, and *Xanthaciura connexionis* (Poudel et al. 2019). Since none of these agents, individually, can control the spread of *A. adenophora* , it is desirable to use multiple agents or means of control to achieve a synergistic effect in an ecosystem‐based weed management strategy.

**FIGURE 1:**
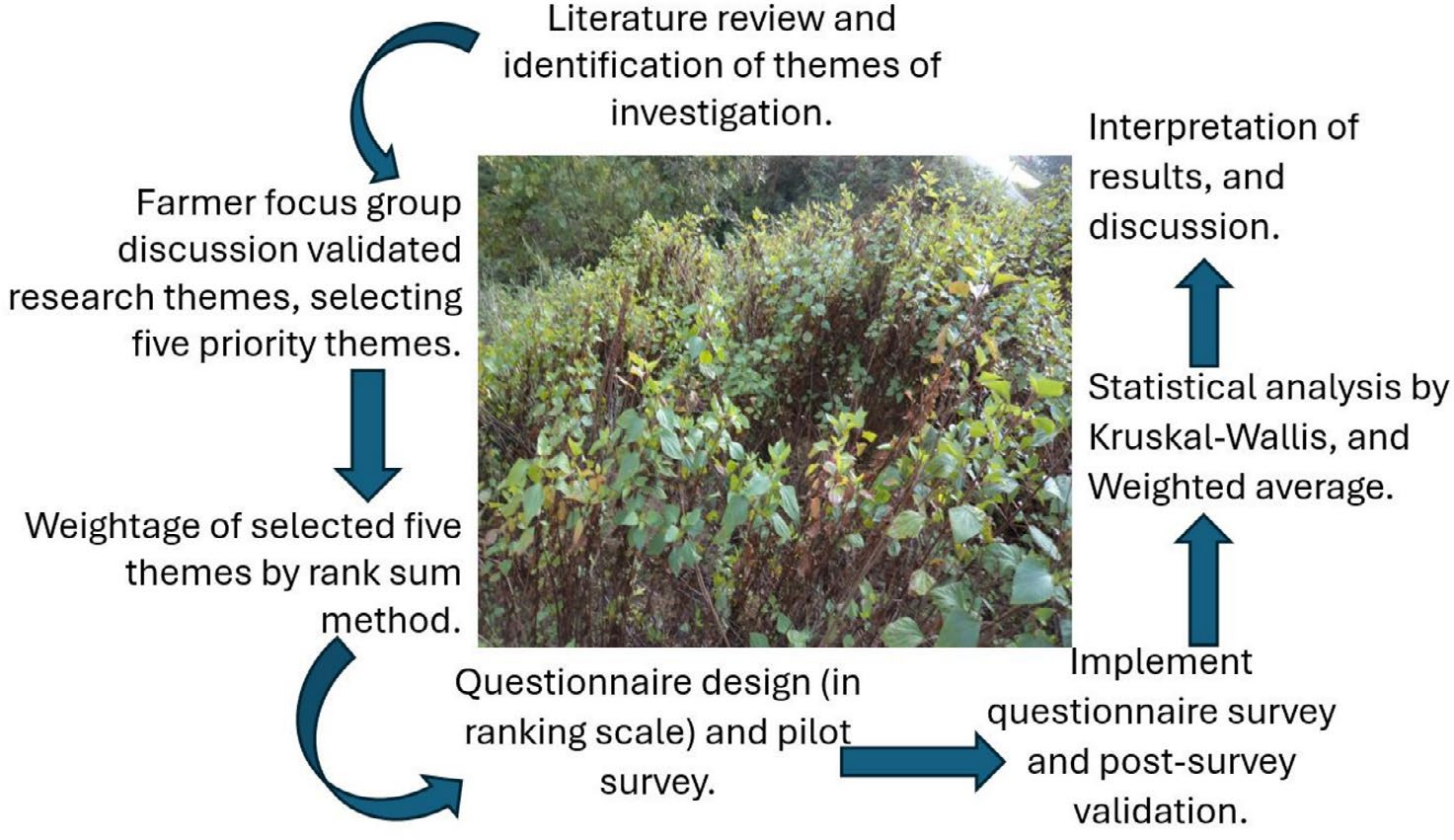
Conceptual diagram of the study.

In Bhutan, *A. adenophora* inhabits roadsides, unused and unmanaged lands, forests, agricultural fields, riverbanks, and open spaces across mid‐tropical to subalpine zones, and is the most aggressively spreading invasive weed in the country (Dorjee et al. 2020; Thinley et al. 2020; Thinley, Banterng, et al. 2022; Lhamo et al. 2023a, 2023b). Its rapid expansion suppresses native biodiversity, undermines crop productivity, and alters soil properties—challenges compounded by rugged terrain, subsistence farming systems, and a shrinking rural workforce due to urban migration (Tshewang et al. 2020). Although the species' biology and control options are well documented, there is a visible gap on how farmers prioritize key aspects like weediness, growth habit, competitive effects, awareness, and control methods in weed management. Socio‐ecological studies are vital for managing invasive species like *A. adenophora* , as they integrate ecological science with community perceptions and cultural values (Wehi et al. 2023). Such approaches highlight Indigenous knowledge and contextualized strategies for prevention and control (Schelhas et al. 2021). Unified frameworks strengthen management decisions (Robertson et al. 2020) as the applied strategies demonstrate socio‐ecological integration into practice (TRCA 2020). Local smallholder farmers possess deep, place‐based knowledge of invasive weeds, yet their valuable insights are rarely tapped for sustainable management strategies (Tesfahunegn et al. 2016; Tippe et al. 2017). Therefore, to fill this gap, we conducted a focus group discussion with village leaders in Kanglung to identify five farmer‐relevant themes, surveyed 91 randomly selected farmers with a five‐point ranking scale, and applied non‐parametric statistical analyses to derive empirically grounded, farmer‐prioritized recommendations for integrated *A. adenophora* management. The findings of this study are expected to inform policies on invasive weed monitoring and management (Weiss et al. 2004), aligning with Bhutan's goal of achieving 100% organic agriculture (Feuerbacher et al. 2018) in the future. Therefore, we hypothesized that the farmers who work in the field and deal with the challenges of this noxious weed would be rich in knowledge through experience in controlling *A. Adenophora* and aim to determine the perspectives of farmers on the impact and management of *A. Adenophora* on agriculture and environment in eastern Bhutan (Figure 1).

## 2 Methodology

### 2.1 Study Area

**FIGURE 2:**
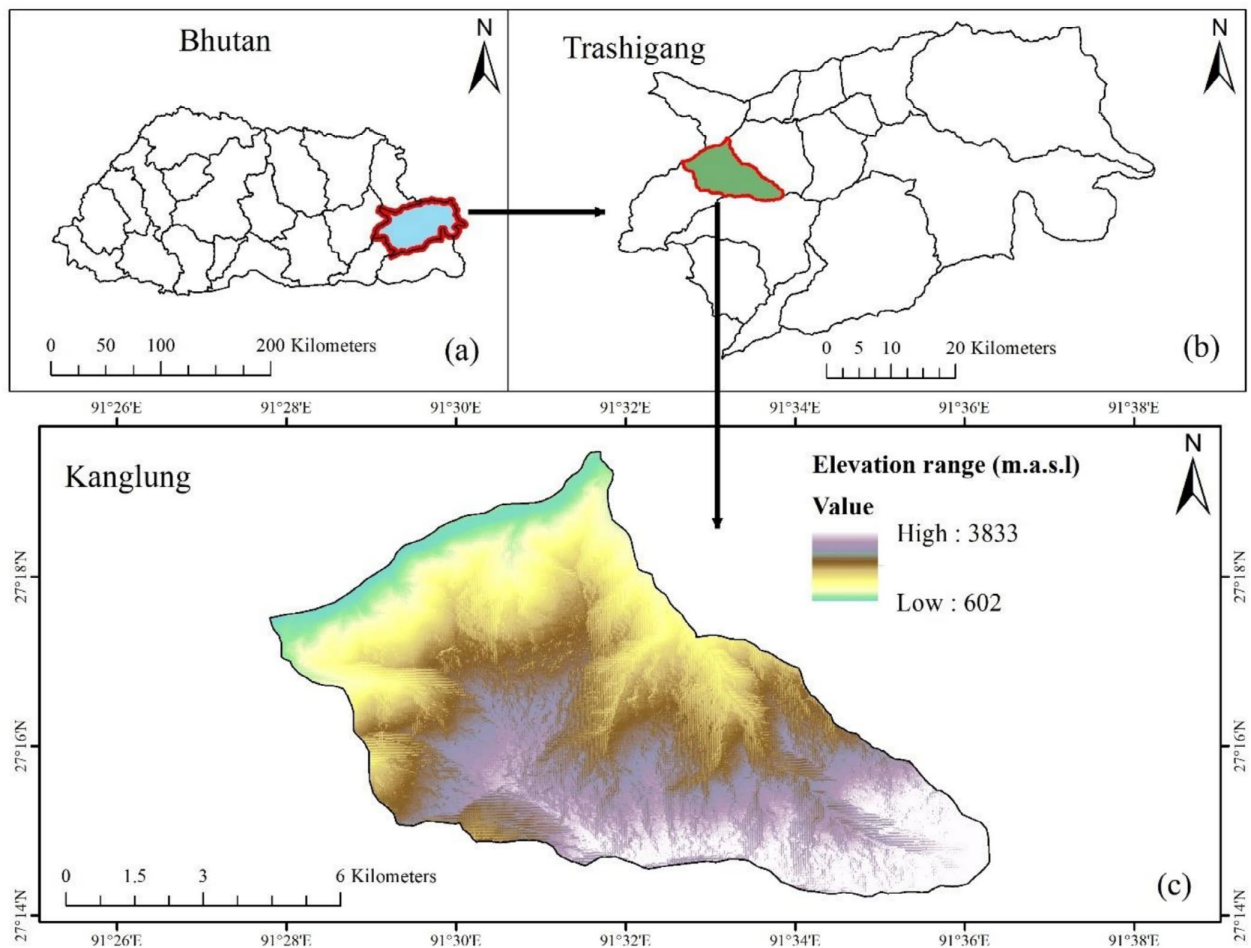
Map of the study area, Kanglung under Trashigang, Bhutan.

Bhutan is a landlocked country nestled in the eastern Himalayas, positioned between India and China. Bhutan is divided into twenty districts (Figure 2a), known locally as *Dzongkhags*, each of which is further subdivided into smaller administrative blocks called *geogs*. The study area for this research is situated within Trashigang district (Figure 2b) of eastern Bhutan marked in green as Kanglung *geog*. Kanglung is geographically located between 27.14° and 27.18° N and 91.26° and 91.38° E with the elevation ranging from 602 to 3833 m.a.s.l. A modern motorable road links Kanglung and Trashigang proper. Additionally, each *geog* is connected by paved bitumen roads, while unsealed farm roads provide access to villages within each *geog*. Kanglung has subtropical climatic conditions with an annual rainfall of 6078 mm, average monthly minimum temperature of 5°C and average monthly maximum temperature of 30°C (NCHM 2018). This climate supports a native dry sub‐tropical broadleaved forest. Predominantly agrarian, farmers in the study area mostly practice subsistence or semi‐subsistence agriculture.

### 2.2 Focus Group Discussions

**FIGURE 3:**
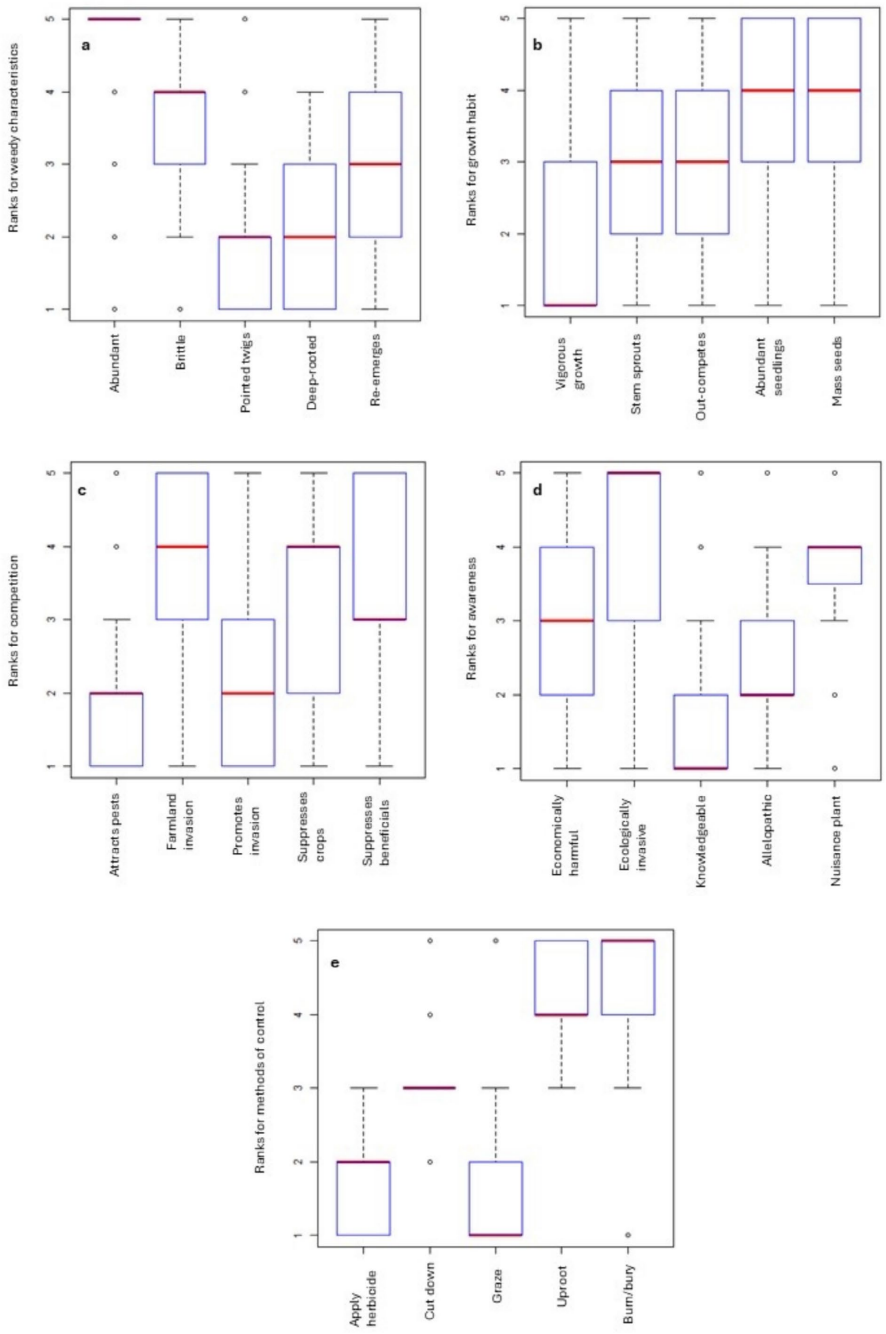
The perception of local people in Kanglung around *A. adenophora* as an invasive plant in their locality as reported by *n* = 91 respondents in the Kanglung *geog* under Trashigang district in Bhutan. The medians are displayed by horizontal (red) lines within the boxes, the 25th and 75th percentiles are represented by the box boundaries; the upper and lower whiskers extend to the largest and smallest values within 1.5 times the interquartile range.

**FIGURE 4:**
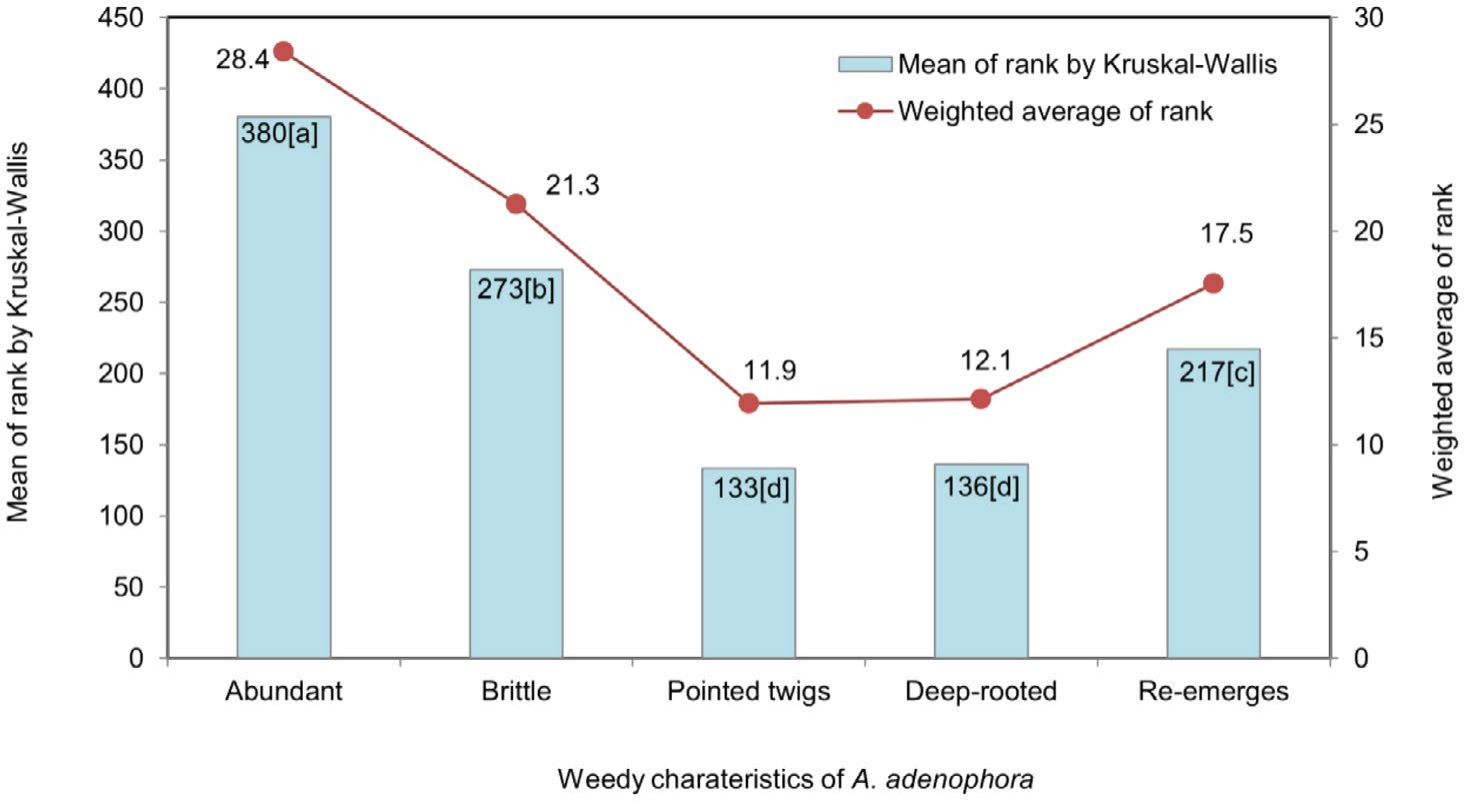
The five sub‐themes included in the *A. adenophora* as a weed are: It is abundantly available (Abundant); it breaks when uprooting (Brittle); twigs from dried biomass pose dangers of piercing (Pointed twigs); root stocks are not removable without plowing (Deep‐rooted); it sprouts from the underground leftover parts (Re‐emerges). Different lower‐case letters in the bars indicate significant differences at *p* < 0.05 according to the Dunn's test.

**FIGURE 5:**
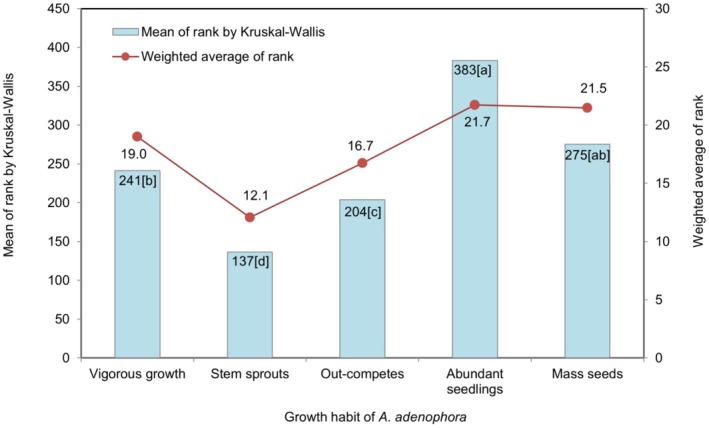
The five sub‐themes included in the *A. adenophora* growth theme: I see plenty of new seedlings in the new season (Abundant seedlings); saplings of *A. adenophora* grow faster than the seedlings of other plants (Vigorous growth); a stem of *A. adenophora* produces a large quantity of seeds (Mass seeds); saplings overtake the growth of other plants (Outcompetes); new plants arise from the aerial plant part (Stem sprouts). Different lower‐case letters in the bars indicate significant differences at *p* < 0.05 according to the Dunn's test.

**FIGURE 6:**
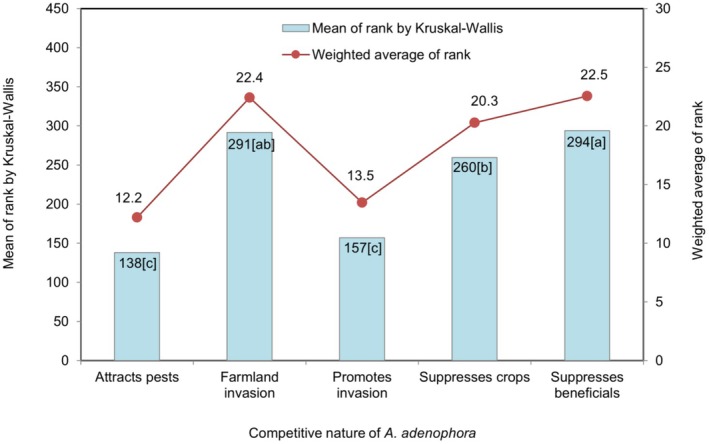
The five sub‐themes included in the competition by *A. adenophora* theme: It grows vigorously in cultivated fields (Farmland invasion); it suppresses the growth of cultivated plants (Suppresses crops); it suppresses the growth of other useful plants (Suppresses beneficials); it attracts harmful insects (Attracts pests); it promotes other weeds to become invasive (Promotes invasion). Different lower‐case letters in the bars indicate significant differences at *p* < 0.05 according to Dunn's test.

**FIGURE 7:**
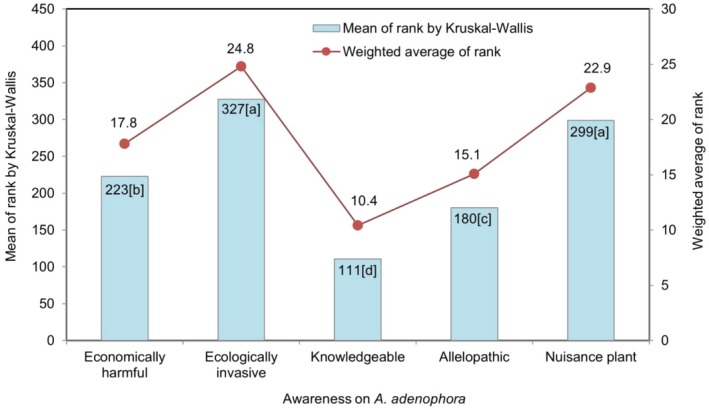
The five sub‐themes included in the awareness on *A. adenophora* theme: Has knowledge about invasive plants (Knowledgeable); *A. adenophora* is invasive (Ecologically invasive); *A. adenophora* is useless (Nuisance plant); *A. adenophora* is economically bad (Economically harmful); *A. adenophora* suppresses the growth of other plants (Allelopathic). Different lower‐case letters in the bars indicate significant differences at *p* < 0.05 according to the Dunn's test.

**FIGURE 8:**
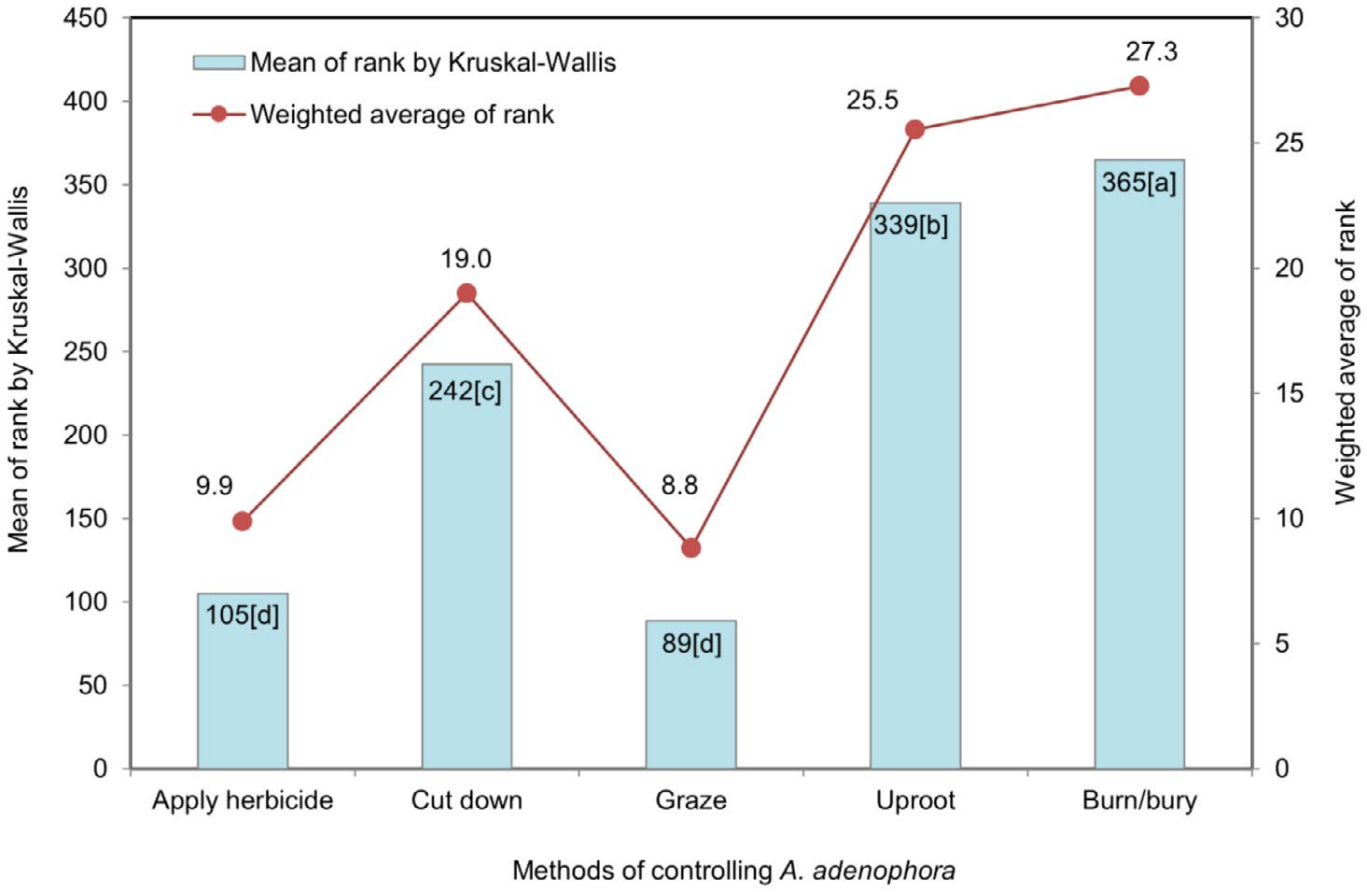
The five sub‐themes included in the methods of *A. adenophora* control theme: Uproot = Uproot; uproot and burn/bury (Burn/bury); cut (Cut down); let livestock graze on it to control (Graze); apply chemicals to kill the plant (Apply herbicide). Different lower‐case letters in the bars indicate significant differences at *p* < 0.05 according to the Dunn's test.

After consulting literature on *A. adenophora* weed, a focus group discussion was conducted to narrow down priority themes relevant to farmers of Kanglung *geog* in the Trashigang district. The focus group discussion held between November and December 2022 composed of local village leaders (*tshogpa*) who are village elders and elder farmers representing their village familiar with the invasive weed *A. adenophora* and its impacts on both the local ecology and economy from within the villages of Kanglung *geog* were invited for the discussion. A total of eight individuals from both genders participated in a one‐day consultation meeting held in the participants' mother tongue (*Tshangla*), with the objective of discussing the prevalence of weeds in each village. Local people identified *A. adenophora* as an “abundantly available, useless plant that grows vigorously and forms dense monocultures”. Nine key themes identified from the review of literature were presented to the group. The nine themes were: *A. adenophora* as a weed; decay of biomass; growth of *A. adenophora* ; control of *A. adenophora* ; usefulness of *A. adenophora* ; competition by *A. adenophora* ; soil quality; awareness on *A. adenophora* ; and methods of control. Upon deliberation and discussion, the group selected and ranked the five most pertinent themes. Subsequently, sub‐themes from the literature were discussed, and five sub‐themes (see Figures 3, 4, 5, 6, 7, 8 for sub‐themes under each theme) were identified for each of the main themes to be included in the questionnaire survey.

### 2.3 Questionnaire Survey

Following the focus group discussions, a semi‐structured questionnaire field survey was designed (see Supporting Information). The questionnaire consisted of five ranking questions (sub‐themes) for the five main *A. adenophora* issues identified in the focus group discussion: weedy characteristics (as a weed), growth habit, competition, awareness, and control methods. Prior to the main survey, a pilot study was conducted with five randomly selected households to test the relevance and sensitivity of the survey. In addition, to ensure consistency in the conduct of the survey, five fourth‐year undergraduates were trained prior to the survey. The questionnaire survey was conducted in Kanglung *geog* (Figure 2c).

A total of 91 farmers were selected using convenient sampling technique (based on the availability and willingness to participate) from the sampling frame and interviewed, which is more than the required sample size of 5% recommended for a representative study (Kotrlik and Higgins 2001). The minimum age of respondents was 25 years old to ensure that the respondents were familiar with and had knowledge of *A. adenophora* . Prior to being interviewed, each farmer was informed and asked to consent, and only those who consented participated in answering the questionnaires. To quantify the qualitative responses for statistical analysis, the five sub‐themes under each of the five priority themes were ranked during the interview based on the respondents' priority on a 5‐point scale (1 = lowest priority, 2 = low priority, 3 = moderate priority, 4 = high priority, and 5 = highest priority). Such scoring is a simple and flexible way of collecting data for a farmer perception study (e.g., Ilbery 1977; Bajgai et al. 2023). Post‐survey cross validation was also conducted with five households selected by stratified random sampling using the same 5‐point response scale to validate the accuracy and consistency of the collected data.

### 2.4 Statistical Analysis

For analysis, five sub‐themes under each of the five priority themes were translated into intuitive words or phrases to enhance interpretability and plotting convenience. Raw data generated was processed using Microsoft Excel, while plots were generated using both Microsoft Excel and the Statistical Tool for Agricultural Research (STAR) Version 2.0.1 (IRRI 2014). The statistical analysis was conducted using IBM SPSS Statistics (Version 22). The Kruskal‐Wallis rank test (the non‐parametric equivalent of one‐way ANOVA) was used to analyze data because the data generated in this study did not meet the assumptions of ANOVA. To determine significant differences among the sub‐themes, Dunn's test was applied for pair‐wise comparisons. This method is more appropriate for analyzing non‐parametric or ordinal data (McKight and Najab 2010; Bajgai et al. 2023). The data collected across five themes—(a) *A. adenophora* as a weed (weedy character), (b) its growth habit, (c) its competitive ability, (d) awareness about *A. adenophora* , and (e) control methods—were first examined using boxplots before being subjected to the Kruskal‐Wallis test. The rank sum method described by Stillwell et al. (1981) was used to determine the weight of each of the five prioritized themes by focus group discussion as

(1) $$W_{i}=\frac{\;n-\mathrm{rj}+1}{⅀\left(n-\mathrm{rk}+1\right)}$$

where *w*_*i*_ is the normalized weight for the *j*th criterion, *n* is the number of criteria, that is the research themes under consideration (*k* = 1, 2, … *n*), *rj* is the rank position of the criterion (theme), and *rk* is the rank position of the *k*th criterion. Each criterion is weighted (*n* − *rk* + 1) and then normalized by the sum of all weights, that is Σ(*n* − *rk* + 1). The weighting percentage was derived by multiplying the value of *W*_*i*_ by 100. Weighted averages were calculated to complement the inferential statistical analysis performed using the Kruskal‐Wallis rank test using a simple formula (Equation 2).

(2) $$X=\frac{\;\sum_{i=1}^{n}\;w_{i}x_{i}\;}{\sum_{i=1}^{n}\;w_{i\;}}$$

where *X* is the weighted average mean, *x*_*i*_ is the multiset of data, and *w*_*i*_ is the weights (1–5) assigned to each of the data sets. The generated Kruskal‐Wallis test by ranks was plotted along with the calculated weighted averages to allow comparisons.

## 3 Results

### 3.1 Demographic Profile of Respondents

A total of 91 farmers participated in the questionnaire survey in Kanglung *geog*, of whom 60.44% were female and 39.56% were male. Most of the farmers (72.53%) were aged between 35 and 55 years, while the remainder were either younger than 35 years (14.29%) or older than 55 years (13.19%). The majority of the respondents had completed either primary school or non‐formal education (41.76%), and 32.97% of the respondents were illiterate. The remaining 25.27% of respondents had completed classes or grades seven to 12 as their highest level of education.

### 3.2 Study Themes and Weightages

The rank scores of each research theme were converted to weightings (or the degree of importance) using the rank sum method. The farmers' top five prioritized research themes on *A. adenophora* were: as a weed (28% weighting), methods of control (24%), competition (20%), growth habit (16%), and awareness (12%).

### 3.3 Statistical Results

#### 3.3.1 Data Distribution

Data distribution by median, spread and skewedness for the five prioritized research themes: as a weed (weedy character) (a), growth habit (b), competition (c), awareness (d), and methods of control (e) was illustrated using box‐and‐whisker plots (Figure 3). There were highly significant differences (*p* < 0.001) between the sub‐themes in all five prioritized themes. A higher mean rank indicates greater priority.

The plots (Figures 4, 5, 6, 7, 8) demonstrate the means of the ranks (by Kruskal–Wallis rank test) and weighted averages of five sub‐themes investigated under the five prioritized research themes of *A. adenophora* .

#### 3.3.2 *Ageratina adenophora* as a Weed

*A. adenophora* as a weed was the highest ranked theme. Of the five sub‐themes in this theme, the abundance of *A. adenophora* was the highest ranked sub‐theme, that is 83.5% farmers ranked it as 5 (data highly skewed to rank 5). This was followed by the tendency of its stems to break while uprooting where 52.7% ranked it 4 (Figure 3a). The third most important sub‐theme was its ability to sprout from underground leftover parts, with 29.7% of the respondents ranking it 3 (data symmetrically distributed). The ease of removing the rootstock and the piercing danger associated with its twigs were of least concern, with 53.8% ranking it 2 and 47.3% ranking it 1, respectively.

The sub‐themes of *A. adenophora* as a weed were highly and significantly different (*p* < 0.001) to each other (Figure 4 and Table 1). The highest mean rank value was for it being abundantly available (380) since the majority (83.5%) of the respondents ranked it 5 (Figure 3a), significantly higher than the other four sub‐themes in the *A. adenophora* as a weed theme. The median of “it breaks when uprooting” lies at 4, with a rank value (273), followed by “sprouts from underground leftover parts” with (217) with its median at 3 (Figure 3a). The lowest rank values were for “root stock is not removable without ploughing” (136), and “twigs from dried biomass pose a danger of piercing” (133), both of which are not significantly different. However, 50% of the respondents ranked the sub‐theme of the “root stock is not removable without plowing” as 2. The values of weighted averages closely follow the means of the ranks cross validating the results in all five prioritized research themes (Figures 4, 5, 6, 7, 8).

**TABLE 1:** Summary of Kruskal–Wallis rank sum test for several dependent samples.

Serial number	Research themes on *A. adenophora*	*n*	df	Chi‐square/test statistics	*p*
a.	Weedy characteristics/(as a weed)	455	4	234.633	< 0.001
b.	Growth habit	455	4	78.945	< 0.001
c.	Competition	455	4	123.278	< 0.001
d.	Awareness	455	4	169.569	< 0.001
e.	Methods of control	455	4	169.569	< 0.001

*Note:* *n* = 455 because there were five major points/issues ranked by 91 farmers (91 × 5 = 455).

#### 3.3.3 Growth of *A. adenophora*

The appearance of plenty of new seedlings of *A. adenophora* in the new season had the highest mean rank (383) as the top sub‐theme under growth theme, followed by mass seed production (275), growing faster than seedlings of other plants (241), overtaking the growth of other plants (204), and new plants arise from the aerial plant part (137) (Figure 5). The two sub‐themes with higher mean rank and weighted averages had the medians as rank 4 (Figure 3b) and were not statistically different (Figure 5). However, pair‐wise comparisons demonstrate the statistical differences among the sub‐themes (Figure 5). The sub‐themes grow faster than the seedlings of other plants and overtake the growth of other plants were not statistically different as both the sub‐themes have the same median rank of 3 (Figure 3b), followed by the sub‐theme arises from aerial plant parts, with the lowest priority with 51.6% of the respondents ranking it 1, with a median 1 (Figure 3b).

#### 3.3.4 Competition by *A. adenophora*

Under the competition theme, suppression of the growth of other useful plants topped the priority sub‐theme, closely followed by the grows vigorously in the cultivated fields sub‐theme with mean ranks 294 and 291, respectively (Figure 6). Both had significantly higher mean ranks than the other three sub‐themes. This trend can be explained by 42.9% and 30.8% of respondents ranked grows vigorously in the cultivated fields and suppresses the growth of other useful plants as 5 (Figure 3c). The sub‐themes it attracts harmful insects (138) and it promotes other weeds to become invasive (157) significantly differed from the other three sub‐themes with pair‐wise comparisons as presented in Figure 6. The sub‐theme suppresses the growth of other useful plants had a mean rank of 260 with 13.2% of respondents ranking it as 5, while 39.6% ranked attracts harmful insects and promotes other plants as 1 with a median of 2 (Figure 3c).

#### 3.3.5 Awareness of *A. adenophora*

Under the awareness theme, 60.4% of the respondents indicated the plant as invasive (Figure 3d) and with a mean rank of 327, it tops the priority information for the awareness theme, followed by the plant being useless with a mean rank of 299; both the sub‐themes significantly differed from the other three (Figure 7). Around 63.4% of the respondents ranked *A. adenophora* as useless with a rank of 4, and 41.4% ranked 3 for the plant being ecologically and economically bad (mean rank = 223) (Figure 3d and Figure 7). Except for the plant being ecologically and economically bad (symmetrical and median = 3), data distribution for other sub‐themes was skewed (Figure 3d). About 73.6% ranked the knowledge of invasive plant as 1 (median = 1), indicating that farmers possessed poor knowledge about what plants are invasive (Figure 3d).

#### 3.3.6 Methods of Control of *A. adenophora*

“Uproot and burn or bury” was the most favored control method with a mean rank 365, significantly higher when compared with the other control methods (sub‐themes) (Figure 8). This is because 64.8% respondents rated it as highest priority (rank 5). Uproot (339) and cutdown (242) were second and third most favored methods of control, respectively when 35.4% respondents ranked uproot as 5 (median) and 70.3% samples ranked cutdown as 3 (median) (Figure 3e). However, livestock grazing (mean rank = 89 and median = 1) and application of chemicals (mean rank = 105 and median = 2) were perceived as the least favored control methods (Figures 3e and 8).

## 4 Discussion

### 4.1 Rationale

The nine themes initially identified for the study through literature reviews included: As a weed (weedy character) (Thinley, Gurung, et al. 2022); growth (Darji et al. 2021; Verma et al. 2023); its biomass recycling (Liu et al. 2022); competitive rigor (Inderjit et al. 2011); awareness, management strategies, and control methods (Poudel et al. 2019); impact on the soil (Darji et al. 2021; Lhamo et al. 2023a; Negi et al. 2023); and usefulness to farmers. These themes were examined by local farmers through a focus group discussion to ensure that they were relevant in the local context. This process was crucial for aligning the selected research themes of investigation with the farmers' knowledge and firsthand experience in managing the challenges posed by *A. adenophora* (Weiss et al. 2004; Tshewang et al. 2020). Further, the focus group discussions confirmed that the weed in question was indeed invasive (Jigme and Bajgai 2019; Dorjee et al. 2020; Thinley et al. 2020; Thinley, Banterng, et al. 2022; Tshewang et al. 2020; Lhamo et al. 2023a, 2023b; Fried et al. 2025). The farmer focus group discussions confirmed the relevance of the research to the local setting by prioritizing the themes or issues as weediness > methods of control > competition > growth habit > awareness, indicating a sequence of priority actions to address the challenges of *A. adenophora* .

### 4.2 *Ageratina adenophora* as a Weed

It is well known that *A. adenophora* is an invasive species around the world (Yu et al. 2016; Poudel et al. 2019; Gong et al. 2022; Fried et al. 2025) and in Bhutan (Thinley et al. 2020; Thinley, Gurung, et al. 2022; Lhamo et al. 2023a, 2023b). Our study confirms its abundant presence in Kanglung, which suggests its spread and invasion of land in eastern Bhutan. Stems of the plant are quite brittle and break easily, a character which makes it difficult when uprooting the plant, rendering it cumbersome to remove completely (Yang et al. 2017); this has been reflected in its high mean rank (273) and its rank of 4 by 52.7% of respondents. Rootstocks (underground leftover parts) are persistent in the soil to give rise to new shoots. This observation suggests the stems may be susceptible to breakage during pulling (Yang et al. 2017); however, it can be removed. As the stems undergo desiccation, they undergo a transformation into thin needles, which have been known to pierce the feet of farmers and the snouts of livestock, effectively deterring them from grazing. This is a significant characteristic that contributes to the proliferation of *A. adenophora* in landscapes. The plant is not favored by animals for browsing, a trait that further fosters its invasive proliferation, in addition to its allelopathic phytotoxic effects (Inderjit et al. 2011; Darji et al. 2021; Wu et al. 2025).

### 4.3 Competition by *A. adenophora*

Intra‐ and inter‐specific competition in biodiversity is a driving force for evolution within a plant's native habitat range. This becomes more pronounced in plants when they develop adaptation strategies to sustain growth in new habitats. The driving force is thought to be a molecular‐level response of the plant to a shift in habitat through gene plasticity (Negi et al. 2023), a trait also triggered by many biophysical changes in the environment (Barrett 2000). Through this strategy, *A. adenophora* can grow in a variety of landscapes and cause diverse impacts to local inhabitants (Negi et al. 2023, 2025; Khatri et al. 2022, 2025a, 2025b; Fried et al. 2025). In Kanglung, farmers reported *A. adenophora* grows vigorously in cultivated fields, agreeing with the modeling result by Thinley et al. (2020), suppressing the growth of cultivated plants through phytotoxic effects as testified by Darji et al. (2021), and suppressing the growth of non‐cultivated useful plants. For example, Lhamo et al. (2023a) reported that herbs are most affected by *A. adenophora* invasion from a field study in western Bhutan. It is evident that three sub‐themes under competition are of higher priority, as their impact on agriculture is direct (Tshewang et al. 2020). According to Negi et al. (2023), the interference phenomenon in question exerts an impact that is not limited to stunting growth but extends to a reduction in yield in crop plants such as rice and soybean. Suppressing the growth of other useful plants is significantly higher than the suppressing of the growth of cultivated plants (Figure 6) primarily due to lower leaf irradiance (Khatri et al. 2022). This also suggests that *A. adenophora* not only impacts farming in cultivated land, but farmers are impacted even more outside of cultivated lands, e.g., with the suppression of fodder grass/herbs and other plants (Tshewang et al. 2020; Lhamo et al. 2023a). It is also possible that *A. adenophora* stimulates other non‐native plant growth as well as the growth of aggressive plants with increased competitive facilitation leading to ecological meltdown (Figure 6). This requires further research.

### 4.4 Growth of *A. adenophora*

Production of large amounts of seed (Khatri et al. 2022) is a key character of invasive plant species as they invade new areas (Barrett 2000). A total of 36.8% farmers ranked this sub‐theme as 5, the highest priority, with responses lying from 3rd to 4th quartiles with a median at rank 4 (Figure 3b). Plenty of new seedlings appearing in new season (mean rank 383 which is the highest mean rank of all sub‐themes in this study) is consistent with the fact that more seeds produce more seedlings, and, as a result of their evolution, lead to challenges in removing this weed (Barrett 2000). The reproductive index, seed number, and weight in *A. adenophora* is a trait influenced by phenotypic plasticity along the elevation and climatic conditions, such that it produces the highest number of seeds at mid‐level elevations of ≈1500 m.a.s.l (Khatri et al. 2022). This result also validates the weedy sub‐theme of *A. adenophora* (Figure 4; mean rank 380) and rank 5 scored by the highest sample (83.5%) for any sub‐themes in the study. Hence, this reflects the cycle of more seeds, more seedlings, higher population densities and propagule, and invasion pressure (Estrada et al. 2016). This supports the prediction by Thinley et al. (2020) that *A. adenophora* is expanding north–south along the longitudes in Bhutan (Dorji et al. 2022). This invasion extension is synonymous with the ecological contention of carrying capacity, when the population explodes, naturally new individuals are pushed to the frontiers facilitated primarily by water currents and other dispersal means of its lightweight seeds (Khatri et al. 2022). Equally important is the growth rate of new seedlings (Figure 5; mean rank 241) and mature stands‐ for the majority of the samples are clustered towards the first quartile (Figure 3b). The weighted average ranks for these sub‐themes show a minimal difference even though Kruskal–Wallis ranks are highly and significantly different with 42.9% and 28.6%, ranking 5 respectively. Similarly, the mean rank and weighted average of seedlings grow faster than seedlings of other plants overtaking those plants growing in proximity. The respondents’ median for this sub‐theme was at rank 3 even though 13.2% ranked it 5 (almost double the portion of rank 5 for the “overtake the growth of other plants” sub‐theme). The worst characteristic of *A. adenophora* is that the plant regrows from the aerial plant parts (mean rank 137), because if the base of cut stems is covered by the wet soil, rooting starts, with no exception. This makes management burdensome.

### 4.5 Awareness of *A. adenophora*

Awareness was the last priority theme selected by the farmers. Appropriate levels of scientific agro‐ecological knowledge and awareness empower farmers to tackle the ecological challenges in agriculture such as efficient management of weeds. Helping farmers with early detection will be a significant step towards effective weed control (Weiss et al. 2004). About 64.4% of respondents ranked invasiveness as the highest priority (rank 5) for invasiveness, followed by the “useless” sub‐theme (median = 4) (Figure 3d). Although the weed was branded “useless”, there may be other uses of the species on farms (e.g., for cattle bedding) or for the extraction of potentially useful chemicals (Weston 2000; Inderjit et al. 2011; Subba and Kandel 2012; Jigme and Bajgai 2019; Darji et al. 2021) or as a source of bio‐energy (Bajgai et al. 2022). Suppressing the growth of other plants is a fourth ranked sub‐theme in this category with a mean rank of 180 (Figure 7) corroborating the findings that landscapes with *A. adenophora* have a much lower diversity index as reported in the Wangdue and Punakha districts in western Bhutan (Lhamo et al. 2023a).

### 4.6 Methods of Controlling *A. adenophora*

In the advanced stages of invasion, eradication through management becomes unfeasible. The problem is exacerbated by the tendency of weeds that grow in cultivated fields to mimic the phenology of crops and evolve to overcome newer control tactics (Barrett 2000). Therefore, it is imperative to ascertain the preferences and priorities of local farmers on adopting an appropriate control method. Burying or burning (mean rank = 365) tops the control method preference closely followed by uprooting (339) (Figure 8). The process of uprooting the plant and either burning or burying it will result in an almost complete elimination of the possibility of regrowth. However, if the root system is not completely removed, the plant may potentially regrow. Cutting down is rated as the third preferred choice for control, but this method is temporary as regrowth quickly occurs, c.f. the subtheme “arises from the aerial plant part” of priority theme “growth”.

It is critical to align control methods with flowering cycles of *A. adenophora* . Therefore, the best time to implement these control methods is before flowering that is in the early spring through summer to avert the weed from seeding (Tripathi et al. 2012; Yang et al. 2017). The application of herbicides to kill the plant is the least preferred control method, with 41.8% of the respondents (rating it as 1 for least preferred) with zero score in rank 5. This suggests that the people of Kanglung are aware of the environmental impact of chemicals. This may also be attributed to the unavailability, cost and distribution of herbicides, given the country's inclination towards organic farming (Tshewang et al. 2020). In contrast, farmers encounter challenges related to an elevated risk of crop yield loss (Negi et al. 2023) due to weed invasion, compounded by the exacerbating labour shortage in rural villages (Tshewang et al. 2020). Livestock grazing to control the weed is the least preferred control option (mean rank = 89). The lower preference for livestock grazing may be attributable to the unpleasant odor emanating from green *A. adenophora* (Jamir et al. 2018), or the potential discomfort experienced by the livestock due to its spiky twigs. A comparison of the results from the control theme reveals that individuals who reported the least knowledge of invasive plant species (Figure 7) exhibited a preference for control methods (Figure 8). This observation suggests that the residents of Kanglung possess an ecological knowledge, which is consistent with contemporary scientific understanding.

### 4.7 Broader Applicability of the Study

The findings and lessons learned from this study lie in its straightforward adoption of its result to other similar agroecological regions for tackling the invasive weed challenges by (1) anchoring management planning in local ecological knowledge, and (2) ensuring researchers and practitioners can replicate this participatory framework in montane, subtropical, or temperate landscapes. Ultimately, the study delivers practicable, farmer‐prioritized actions that align well with scientific control strategies and community needs. This not only enhances broader adoption potential but also supports sustainable outcomes, providing a scalable model for integrated weed management in diverse agro‐ecological contexts. In addition, the findings highlight important implications for invasive species management decisions and suitable policy directions; such an approach not only improves management of *A. adenophora* in Kanglung but also provides a model for addressing other invasive weeds across ecosystems.

## 5 Conclusion

Farmers of Kanglung, Eastern Bhutan prioritize *A. adenophora* as a weed, its growth, competitive rigor, its awareness, and control methods as pertinent themes to garner a holistic understanding to help manage *A. adenophora* invasion. The results of this research show that farmers of Kanglung prioritize integrated and environmentally friendly weed management as opposed to easy‐to‐use and fast‐acting herbicides.

Therefore, this study addresses the knowledge gap; the lack of systematic, quantified insight into farmers' priority strategies for invasive‐weed management. This demonstrates a replicable, participatory framework that can be applied in other agroecological regions facing similar challenges. By integrating scientific control recommendations with local ecological knowledge, the study not only enhances adoption potential in entire eastern Bhutan but also offers a scalable model for integrated, sustainable weed management in comparable national and regional agroecological contexts. It is vital to keep track of such study's policy implications, more importantly the economic and ecological viability of farmers' priority centric management decisions, to authenticate and strike a balanced integration of local ecological knowledge with modern science based invasive species management.

## Funding

This work was supported by Sherubtse College, Royal University of Bhutan (Grant 15 (3)‐SC/DRIL/2022/10), 22nd May 2022.

## Consent

The basic principles of The Belmont Report 1978 (Biomedical and Research 1978) such as only interviewing consenting participants and voluntary participation were the basis for participation in this study. Prior to an interview, each farmer was briefed and asked for consent and only farmers who consented were included in the focus group discussions and responding to questionnaires. Written permissions were sought from the block (*geog*) head before entering the villages for the focus group discussions and questionnaire surveys.

## Conflicts of Interest

The authors declare no conflicts of interest.

## Supporting information


**Data S1:** pei370110‐sup‐0001‐Supinfo1.pdf.

**Data S2:** pei370110‐sup‐0002‐Supinfo2.pdf.

## Data Availability

The data that supports the findings of this study are available in the Supporting Information of this article, and the additional data may be provided upon request to the corresponding author.
