# Supplementary material for: Participatory Ecological Assessment of Farmer Perspectives on Management of Invasive Ageratina adenophora in Eastern Bhutan
Source: Plant Environ Interact. 2026 Jan 4;7(1):e70110. doi: 10.1002/pei3.70110 (PMC12766076; doi:10.1002/pei3.70110)
Supplement: Supplementary file 1 — Data S1: pei370110‐sup‐0001‐Supinfo1.pdf. [file PEI3-7-e70110-s001.pdf]

Biometric information

Gender: Male [1] Female [2]

Age: 25-35. 36-45. 46-60. >60.

Education: Not attained [0] NFE+Pry [1] 7-10 [2] 11-12 [3]

Technical questions

Ranking question for measuring the impact of *A. adenophora* in Kanglung geog: Agro-Ecosociological method (rank the statements from 1-5, 1=least important, 5=most important).

1. Adenophora as a weed
   - It is abundantly available: abundant
   - It breaks when uprooting: brittle
   - Root stock is not removable without ploughing: deep rooted
   - Twigs from dried biomass poses danger of piercing: pointed twigs
   - It sprouts from the underground leftover parts: re-emerges
2. Growth of *A. adenophora*
   - I see plenty of new saplings in the new season: abundant seedlings
   - Saplings of *A. adenophora* grow faster than the saplings of other plants: vigorous growth
   - A stem of *A. adenophora* produces a mass of seeds: mass seeds
   - Saplings overtake the growth of other plants: outcompetes
   - New plant arises from the aerial plant part: stem sprouts
3. Competition by *A. adenophora*
   - It grows vigorously in the cultivated field: farmland invasion
   - It suppresses the growth of cultivated plants: suppresses crops
   - It suppresses the growth of other useful plants: suppresses beneficials
   - It attracts harmful insects: attracts pests
   - it promotes other weeds to become invasive: promotes invasion
4. Awareness on *A. Adenophora*
   - Has knowledge about invasive plants: knowledgeable
   - *A. adenophora* is invasive: ecologically invasive
   - *A. adenophora* is useless: nuisance plant
   - *A. adenophora* is economically bad: economically harmful
   - *A. adenophora* suppresses the growth of other plants: allelopathic
5. Methods of control
   - Uproot: uproot
   - Uproot and burn/bury: burn/bury
   - Cut: cut down
   - Let livestock to graze on it to control: graze
   - Apply chemicals to kill the plant: apply herbicides
