# Supplementary material for: Participatory Ecological Assessment of Farmer Perspectives on Management of Invasive Ageratina adenophora in Eastern Bhutan
Source: Plant Environ Interact. 2026 Jan 4;7(1):e70110. doi: 10.1002/pei3.70110 (PMC12766076; doi:10.1002/pei3.70110)
Supplement: Supplementary file 2 — Data S2: pei370110‐sup‐0002‐Supinfo2.pdf. [file PEI3-7-e70110-s002.pdf]

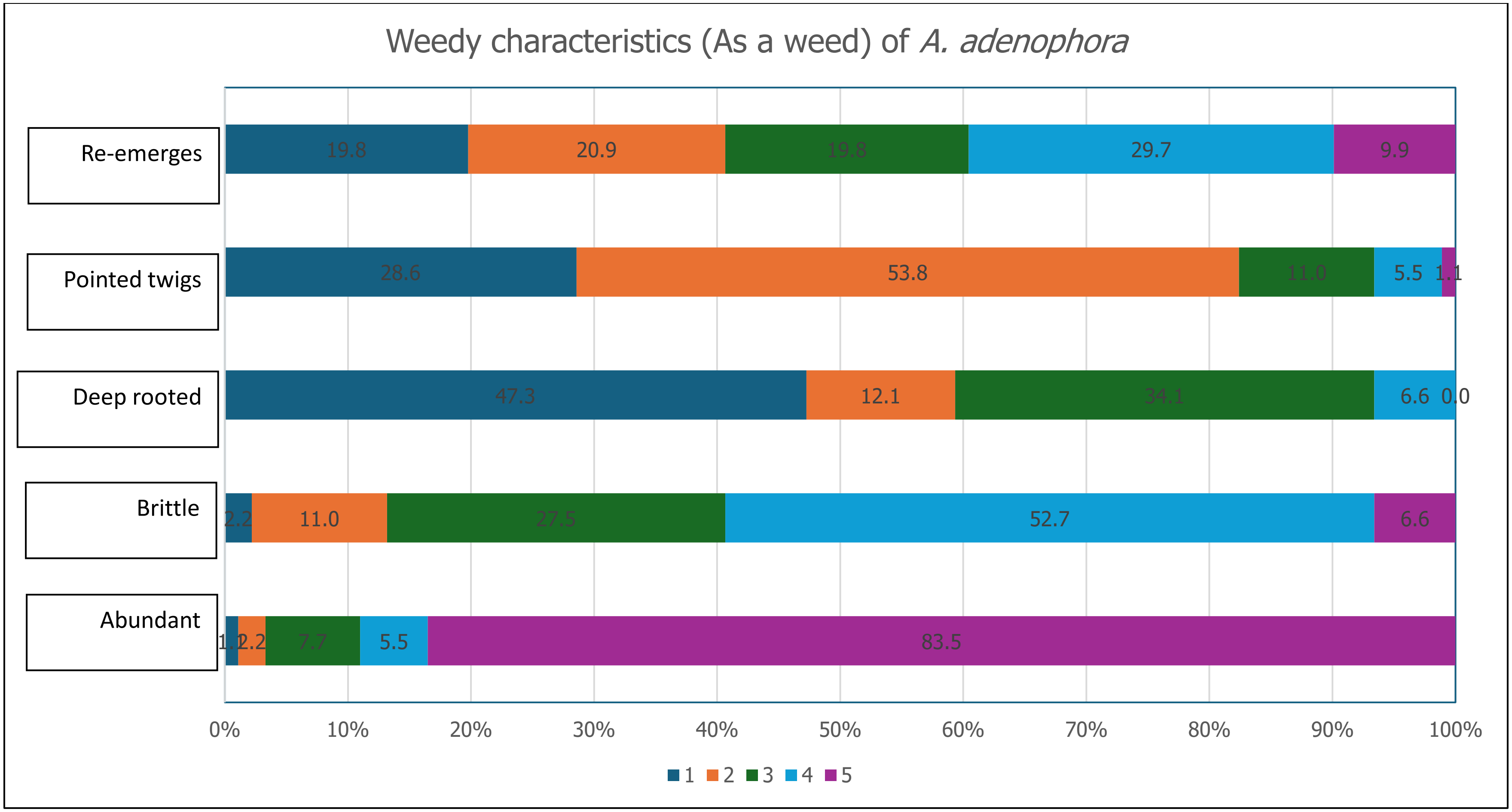

Weedy characteristics (As a weed) of A. adenophora
Re-emerges
19.8
20.9
19.8
29.7
9.9
Pointed twigs
28.6
53.8
11.0
5.5
1.1
Deep rooted
47.3
12.1
34.1
6.6
0.0
Brittle
2.2
11.0
27.5
52.7
6.6
Abundant
1.1
2.2
7.7
5.5
83.5
0%
10%
20%
30%
40%
50%
60%
70%
80%
90%
100%
1
2
3
4
5

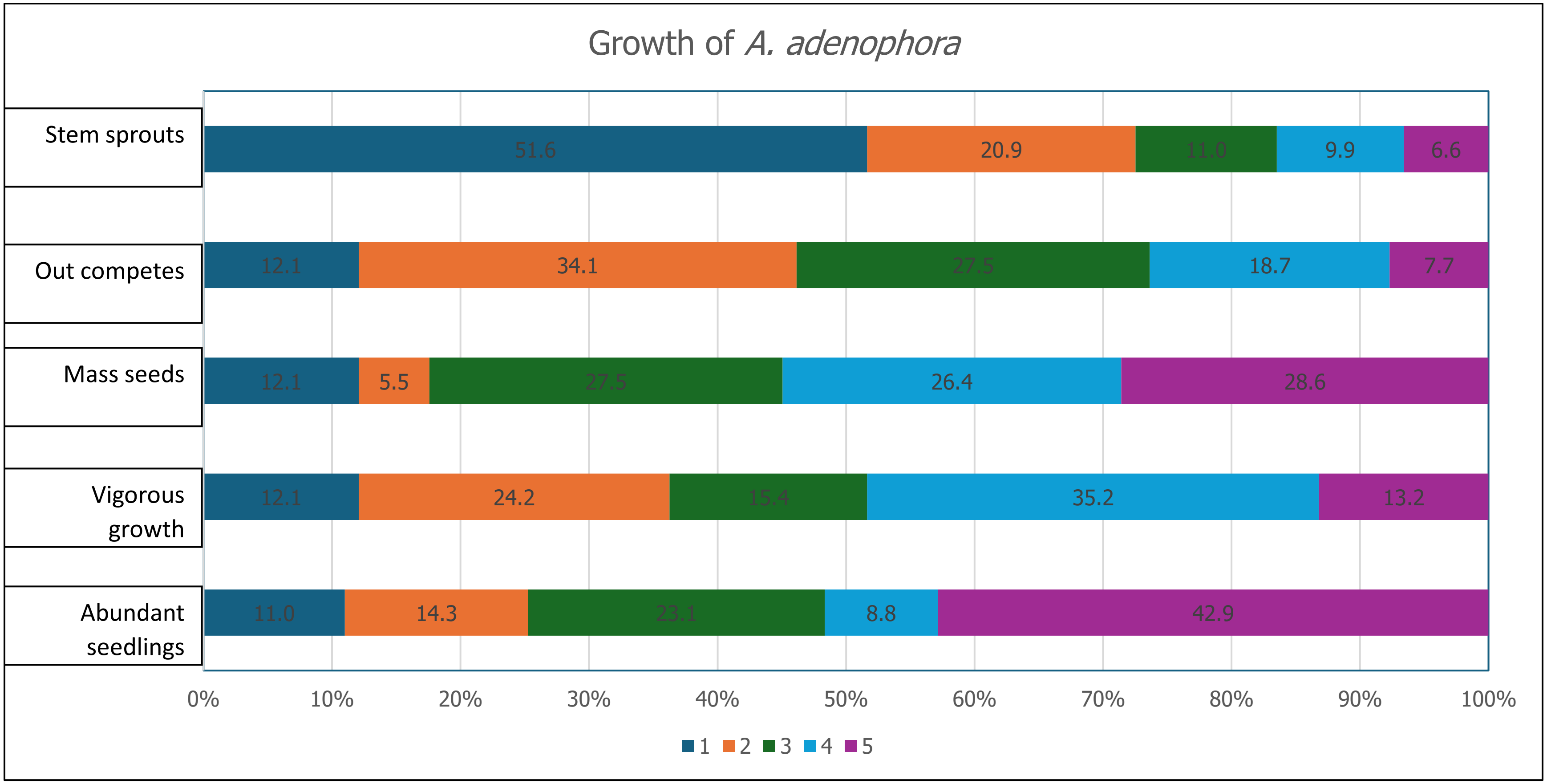
Growth of A. adenophora
Stem sprouts
Out competes
Mass seeds
Vigorous growth
Abundant seedlings
51.6
20.9
11.0
9.9
6.6
12.1
34.1
27.5
18.7
7.7
12.1
5.5
27.5
26.4
28.6
12.1
24.2
15.4
35.2
13.2
11.0
14.3
23.1
8.8
42.9
0%
10%
20%
30%
40%
50%
60%
70%
80%
90%
100%
1
2
3
4
5

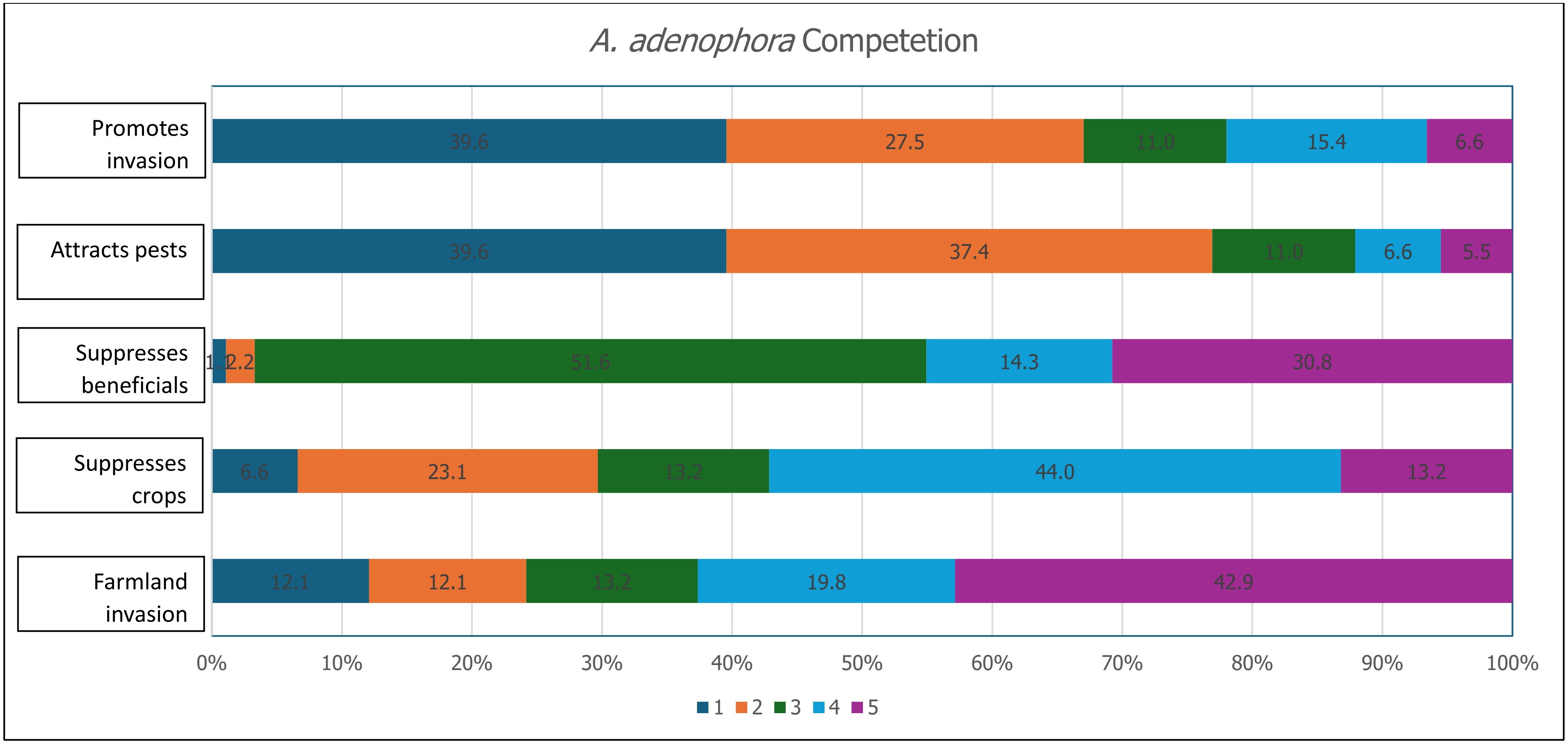
A. adenophora Competetion
Promotes invasion
Attracts pests
Suppresses beneficials
Suppresses crops
Farmland invasion
39.6
27.5
11.0
15.4
6.6
39.6
37.4
11.0
6.6
5.5
1.1
2.2
51.6
14.3
30.8
6.6
23.1
13.2
44.0
13.2
12.1
12.1
13.2
19.8
42.9
0%
10%
20%
30%
40%
50%
60%
70%
80%
90%
100%
1
2
3
4
5

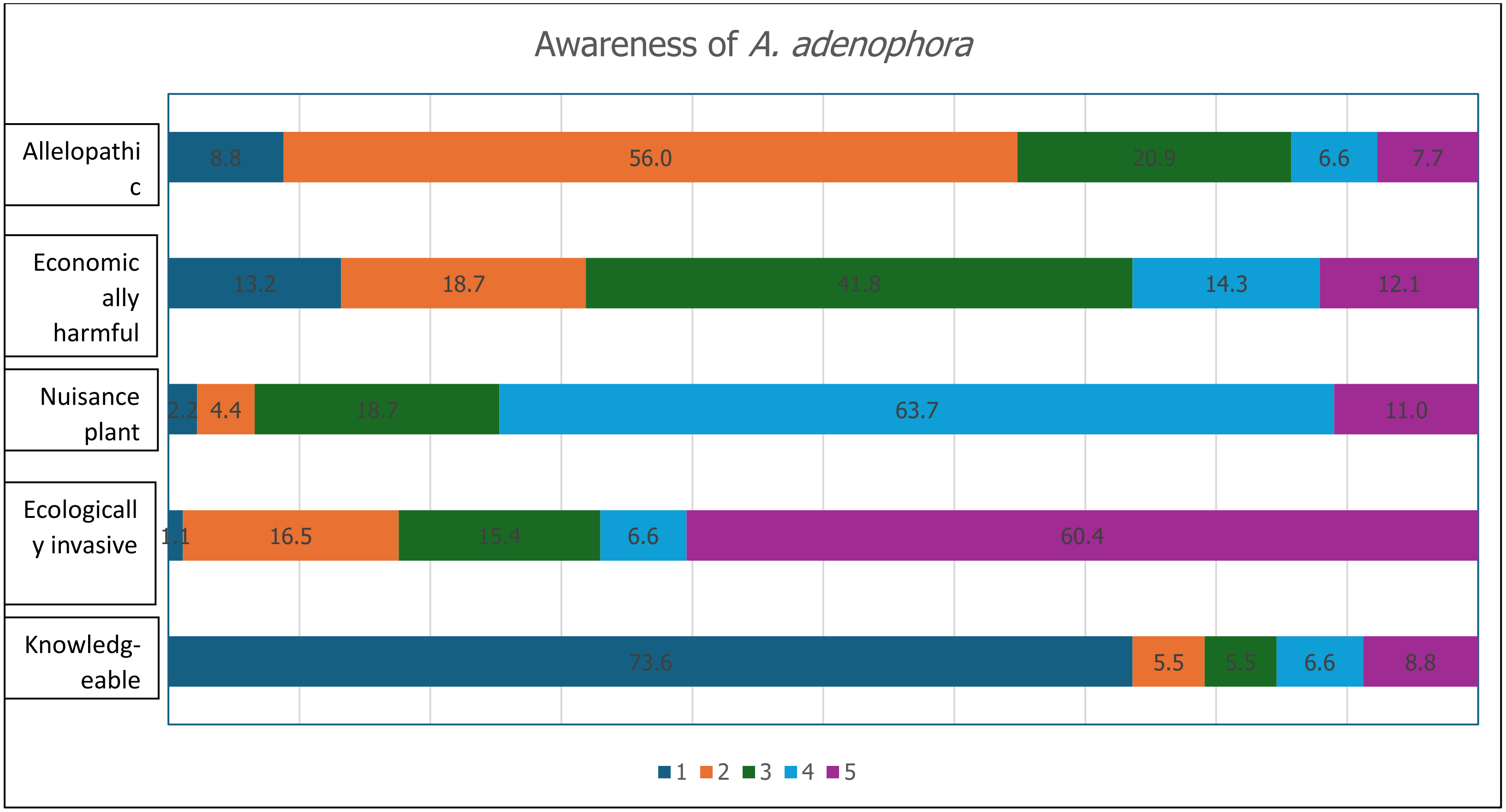
Awareness of *A. adenophora*
Allelopathic
Economically harmful
Nuisance plant
Ecologically invasive
Knowledg-eable
8.8
56.0
20.9
6.6
7.7
13.2
18.7
41.8
14.3
12.1
2.2
4.4
18.7
63.7
11.0
1.1
16.5
15.4
6.6
60.4
73.6
5.5
5.5
6.6
8.8
1
2
3
4
5

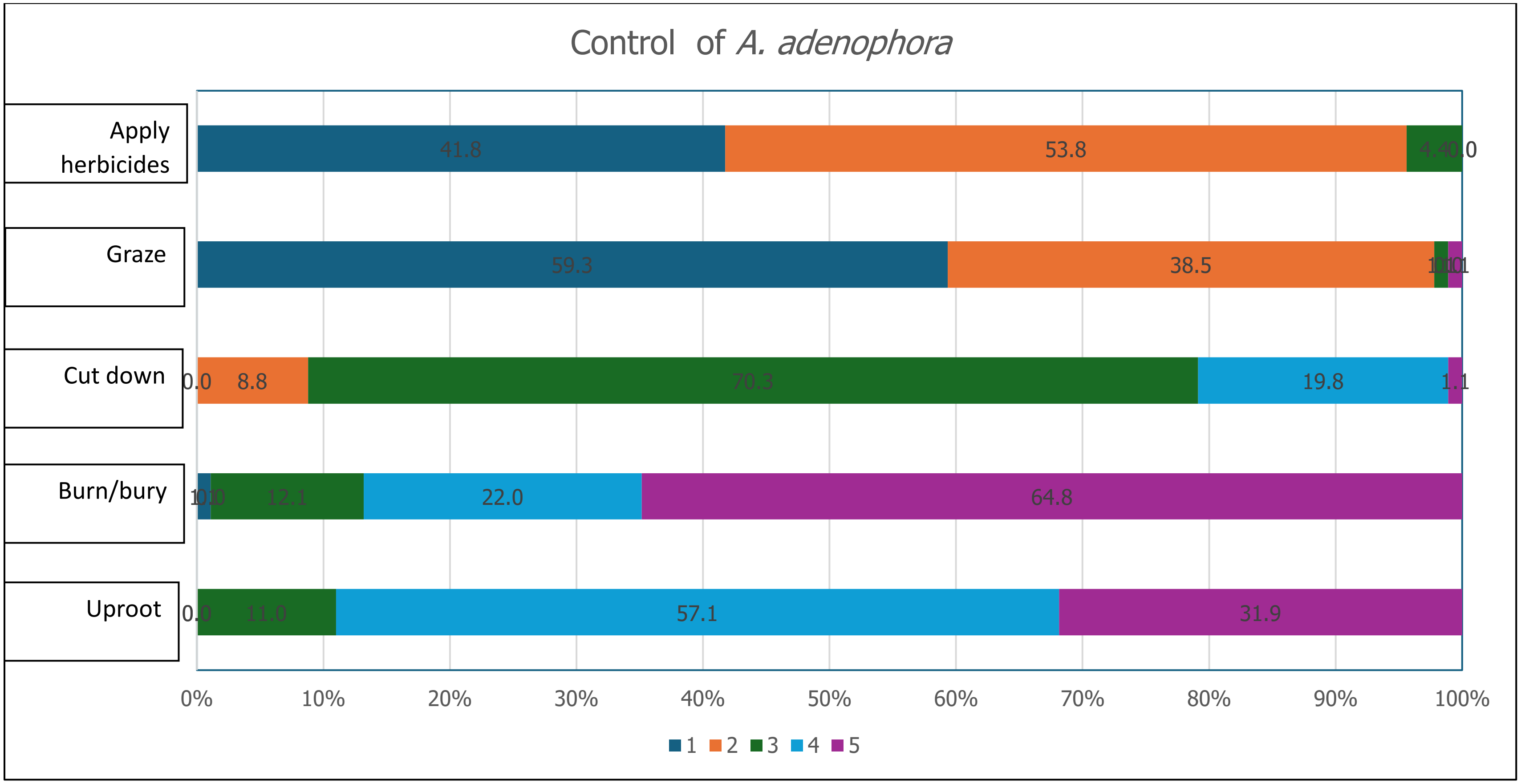

Control of *A. adenophora*
Apply herbicides
Graze
Cut down
Burn/bury
Uproot
41.8
53.8
4.4
0.0
59.3
38.5
0.0
8.8
70.3
19.8
1.1
12.1
22.0
64.8
0.0
11.0
57.1
31.9
0%
10%
20%
30%
40%
50%
60%
70%
80%
90%
100%
1
2
3
4
5
